# Lymphedema Is Associated with Mitral Annular Abnormalities—A Three-Dimensional Speckle-Tracking Echocardiographic Analysis from the MAGYAR-Path Study

**DOI:** 10.3390/biomedicines14092062

**Published:** 2026-09-14

**Authors:** Attila Nemes, Nóra Ambrus, Lajos Kemény, Győző Szolnoky

**Affiliations:** 1 Department of Medicine, Albert Szent-Györgyi Medical School, University of Szeged, H-6720 Szeged, Hungary; ambrusnora@gmail.com; 2 Department of Dermatology and Allergology, Albert Szent-Györgyi Medical School, University of Szeged, H-6720 Szeged, Hungary; kemeny.lajos@med.u-szeged.hu (L.K.); szolnoky.gyozo@med.u-szeged.hu (G.S.)

**Keywords:** lymphedema, mitral annulus, three-dimensional, echocardiography

## Abstract

**Introduction:** Lymphedema is a chronic, progressive disorder characterized by the excessive accumulation of lymphatic fluid within the interstitial compartment resulting from impaired lymphatic transport. Consequently, the primary aim of the present study was to evaluate three-dimensional speckle-tracking echocardiography (3DSTE)-derived mitral annular (MA) morphology and function in patients with lymphedema, and to compare these parameters with those of age- and sex-matched healthy controls. Associations between MA parameters and regional LV strains were also assessed. Furthermore, it was investigated whether one-hour use of medical compression stockings (MCSs) exerts acute effects on MA geometry and function and regional LV strains in lymphedema. **Methods:** The study included 24 patients with lymphedema (mean age: 48.0 ± 11.2 years, 23 females) without known cardiovascular symptoms. Their results were compared to those of 45 healthy individuals (mean age: 42.1 ± 13.9 years, all females) with a similar mean age value and gender ratio. Patients and controls had no traditional cardiovascular risk factors. All patients and control subjects underwent conventional M-mode and two-dimensional Doppler echocardiography extended with 3DSTE. **Results:** At rest, radial and circumferential strains (LV-RS and LV-CS, respectively) tended to increase across most LV regions, except for basal LV-RS. Conversely, longitudinal contraction, represented by LV longitudinal strain (LV-LS) was reduced in the basal LV region but tended to increase in the midventricular and apical regions. This distinct contractile pattern was accompanied by reduced end-diastolic MA dimensions, whereas end-systolic parameters remained unchanged, consequently leading to impaired MA sphincter-like function. While most regional LV strains shifted toward control values following MCS use—except for basal and apical LV-RS and midventricular and apical LV-LS—end-diastolic MA dimensions simultaneously demonstrated a similar trend, culminating in an improvement in MA functional properties. **Conclusions:** The presented exploratory findings suggest that lymphedema may be associated with alterations in end-diastolic MA dimensions and a consequent impairment of MA function, which appear to be accompanied by specific regional LV functional abnormalities. Furthermore, one-hour MCS use was observed to exert acute, directional shifts in these imaging-derived surrogate parameters.

## 1. Introduction

Lymphedema is a chronic, progressive disorder characterized by the excessive accumulation of lymphatic fluid within the interstitial compartment resulting from impaired lymphatic transport [1,2,3,4]. The intensive management of lymphedema relies on complex decongestive physiotherapy, which encompasses meticulous skincare, manual lymphatic drainage, multilayer short-stretch bandaging, specialized exercises, and, in selected cases, surgical intervention while the maintenance phase is usually based on the use of elastic compression garments [2,3,4]. Potential lymphedema-associated cardiovascular alterations remain poorly characterized in the current literature. Three-dimensional (3D) speckle-tracking echocardiography (3DSTE) has emerged as a novel, non-invasive imaging technique capable of simultaneously quantifying mitral annular (MA) dimensions and dynamics as well as regional left ventricular (LV) function [5,6,7,8,9,10,11,12]. Consequently, the primary aim of the present study was to evaluate 3DSTE-derived MA morphology and function in patients with lymphedema, and to compare these parameters with those of age- and sex-matched healthy controls. The study also aimed to examine the associations between MA parameters and regional LV strains. Furthermore, we investigated whether a 1-h application of medical compression stockings (MCSs) exerts acute effects on MA geometry, MA function, and regional LV strains in lymphedema.

## 2. Materials and Methods

### 2.1. Patient Population

This study included 24 patients with stage 2 bilateral lower-extremity lymphedema (mean age: 48.0 ± 11.2 years; body mass index: 27.2 ± 2.4 kg/m^2^; 23 females) without known cardiovascular disease or symptoms, recruited within the framework of the **MAGYAR-Path Study** (**M**otion **A**nalysis of the heart and **G**reat vessels b**Y** three-dimension**A**l speckle-t**R**acking echocardiography in **Path**ological cases). The MAGYAR-Path Study was designed to assess the clinical utility, as well as the diagnostic and prognostic relevance, of 3DSTE-derived metrics across various pathological conditions (notably, the acronym “MAGYAR” translates to Hungarian). Regarding the etiology of secondary lower-extremity lymphedema, 2 cases were attributed to treatment for malignant melanoma, 6 to gynecological cancer treatment, 2 to bacterial cellulitis, and 15 to chronic venous insufficiency. Bilateral lower extremity lymphoscintigraphy was performed in 9 individuals, whereas color duplex ultrasonography was performed in all participants with suspected venous disease. All patients with lymphedema were referred for echocardiographic evaluation from the Phlebolymphology Unit of the Department of Dermatology and Allergology at the University of Szeged. Findings in the lymphedema cohort were compared with those of a control group consisting of 45 healthy individuals (mean age: 42.1 ± 13.9 years; all females) matched for age and sex distribution. Neither patients with lymphedema nor healthy controls had traditional cardiovascular risk factors. All study participants underwent standard M-mode and two-dimensional (2D) Doppler echocardiography, which was supplemented by 3DSTE acquisition at the conclusion of the protocol. Image analysis was performed by an observer blinded to the clinical status. The trial protocol received formal ethical clearance from the Institutional and Regional Human Biomedical Research Committee of the University of Szeged, Hungary (approval number: 71/2011, latest approval 17 March 2025), and all enrolled participants provided written informed consent.

### 2.2. M-Mode and Two-Dimensional Doppler Echocardiography

Prospective 2D Doppler echocardiographic assessments were performed using a Toshiba Artida™ ultrasound system (Toshiba Medical Systems, Tokyo, Japan) equipped with a 1–5 MHz PST-30BT phased-array transducer (Toshiba Medical Systems, Tokyo, Japan). Conventional echocardiographic parameters were evaluated in accordance with contemporary guidelines. Color Doppler echocardiography was used to assess the severity of valvular regurgitation. Early (E) and late (A) diastolic mitral inflow velocities were determined via pulsed-wave Doppler echocardiography. M-mode echocardiography was used for determination of MA plane systolic excursion (MAPSE) [13].

### 2.3. Three-Dimensional Speckle-Tracking Echocardiography

For 3DSTE data acquisition, the same Toshiba Artida™ system (Toshiba Medical Systems, Tokyo, Japan) was used with a PST-25SX matrix-array transducer (Toshiba Medical Systems, Tokyo, Japan). Complete 3D datasets were acquired from the apical window during a single breath-hold at a stable R-R interval on the electrocardiogram (ECG). The digitally acquired 3D datasets comprised six wedge-shaped subvolumes, which were automatically merged by the system software into a comprehensive full-volume dataset. Offline 3D analysis was subsequently performed using the 3D Wall Motion Tracking software (version 2.7, UltraExtend, Toshiba Medical Systems, Tokyo, Japan) [5,6,7,8,9,10].

**Figure 1:**
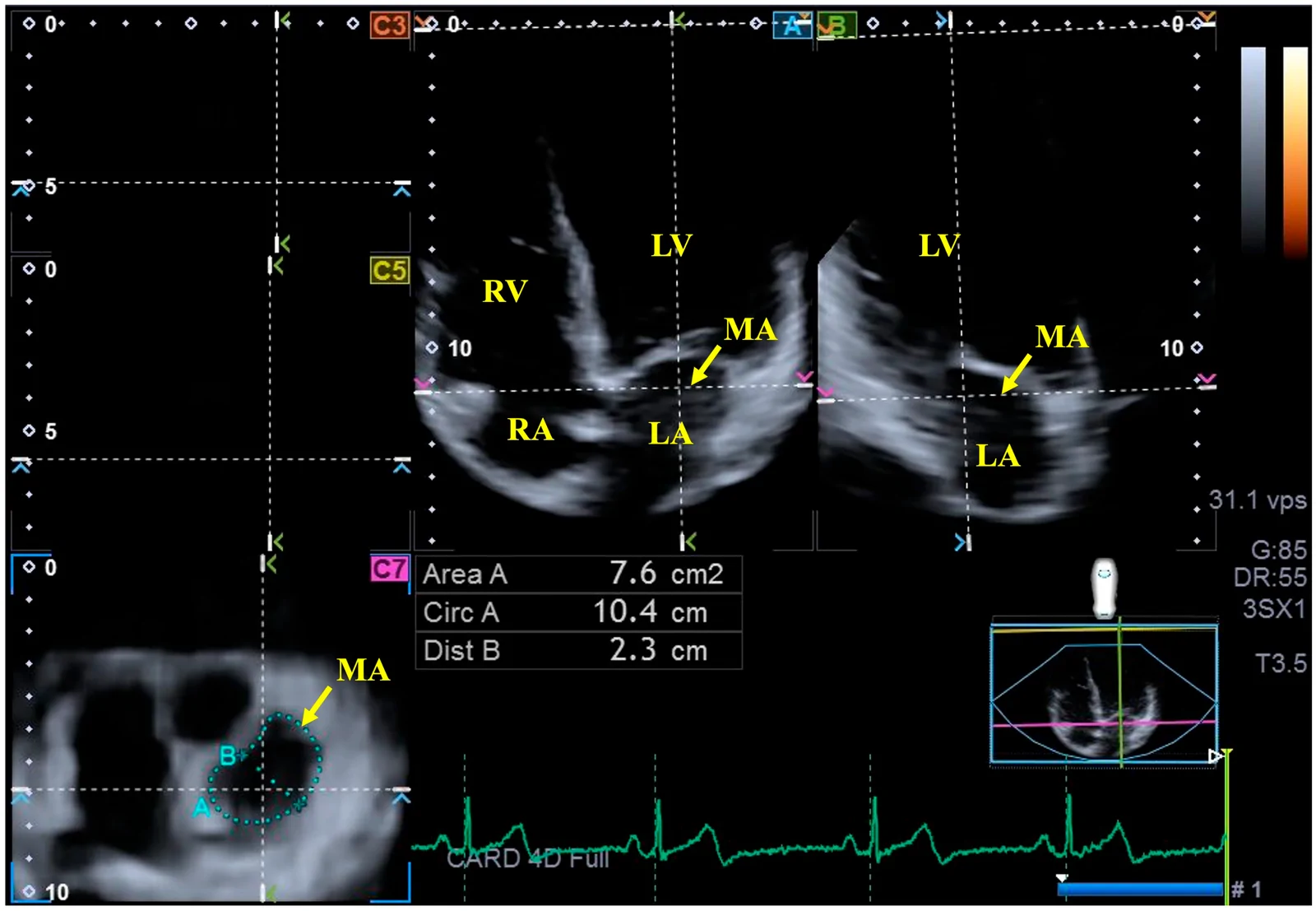
A three-dimensional speckle-tracking echocardiography-derived full-volume dataset displaying the mitral annulus in a representative case: (A) apical four-chamber view, (B) apical two-chamber view, and (C7) a short-axis cross-sectional view at the level of the mitral annulus, optimized using the apical four- and two-chamber long-axis views. The yellow arrow delineates the mitral annular plane across both the long-axis (A, B) and short-axis (C7) projections. Abbreviations: Area = mitral annular area, Circ = mitral annular perimeter, Dist = mitral annular diameter, LA = left atrium, LV = left ventricle, MA = mitral annulus, RA = right atrium, RV = right ventricle.

The software automatically generated apical four-chamber (AP4CH) and two-chamber (AP2CH) long-axis views, alongside three short-axis views at various ventricular levels. Following the optimization of the lateral and septal MA endpoints in both the AP4CH and AP2CH views, MA dimensions were quantified in the C7 short-axis view at both end-systole and end-diastole [11] (Figure 1):

- MA diameter (MAD) was defined as the length of a perpendicular line drawn from the peak of the MA curvature to the midpoint of the straight MA border.
- MA area (MAA) was calculated via planimetry.
- MA perimeter (MAP) was also calculated via planimetry.

To evaluate MA dynamics, the following functional indices were calculated:

- MA fractional shortening (MAFS, %) = (end-diastolic MAD − end-systolic MAD)/(end-diastolic MAD) × 100.
- MA fractional area change (MAFAC, %) = (end-diastolic MAA − end-systolic MAA)/(end-diastolic MAA) × 100.

**Figure 2:**
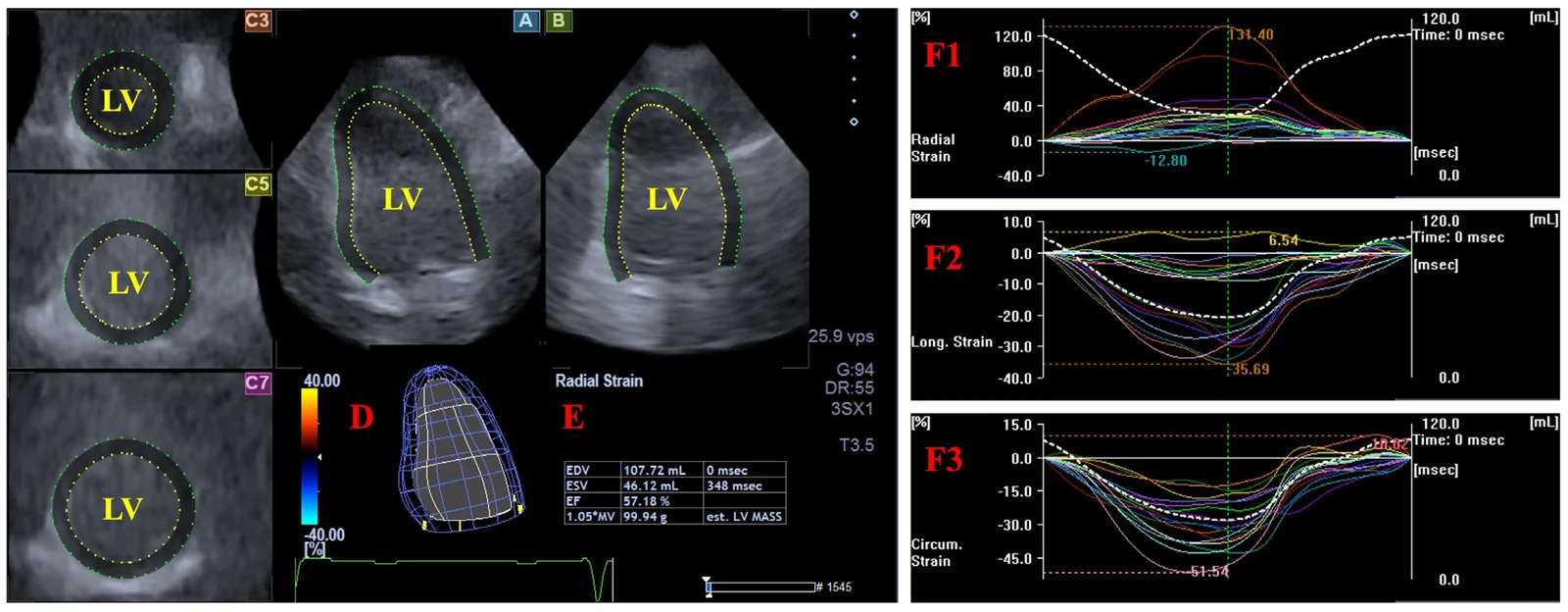
Three-dimensional (3D) speckle-tracking echocardiographic measurement of left ventricular (LV) strains in a representative case. Apical four-chamber (A) and two-chamber (B) long-axis views are shown alongside apical (C3), mid-ventricular (C5), and basal (C7) short-axis views, all of which were automatically reconstructed from the 3D echocardiographic dataset. Panel (D) illustrates the 3D cast of the LV, while panel (E) displays the corresponding LV volumes and ejection fraction. Time–LV radial, longitudinal, and circumferential global (white) and segmental (colored) strain curves, together with the time–LV volume change (dashed white) curve, are alsopresented (F1–F3). Abbreviations: LV = left ventricle, EDV = end-diastolic volume, ESV = end-systolic volume, EF = ejection fraction.

Following the MA measurements, the septal and lateral margins of the LV-MA junction and the endocardial surface of the LV apex were manually delineated by the observer. Following automated border detection and sequential analysis, a virtual 3D cast of the LV was generated. From this model, regional (basal, mid-ventricular, and apical) unidirectional/unidimensional LV strains were determined, calculated as the mean of the corresponding segmental values [12,13,14] (Figure 2):

- Radial strain (RS): representing myocardial thickening/thinning.
- Circumferential strain (CS): representing myocardial narrowing/widening.
- Longitudinal strain (LS): representing myocardial shortening/lengthening.

### 2.4. Experimental Study Protocol

Following the baseline 2D echocardiographic evaluation, all lymphedema patients underwent a 3DSTE examination. Subsequently, MCS were applied for 60 min. Immediately prior to the removal of the MCS, a repeat echocardiographic assessment was performed. A particular strength of the present study was the use of individually tailored MCS for each patient. During the 1-h compression period, patients were instructed to refrain from physical exercise, prolonged standing, sitting with extended legs, and eating or drinking. Environmental conditions were strictly maintained at a room temperature of 21–22 °C and a relative humidity of 45–50%. Compression therapy was performed using flat-knitted black Bauerfeind VenoTrain CuraFlow stockings (Bauerfeind, Zeulenroda, Germany; compression class 2, 23–32 mmHg) composed of 73% polyamide and 27% elastane. Interface pressure between the skin and the compression fabric was monitored at the B1 anatomical landmark in a standing position using a Picopress device (Microlab Elettronica, Nicolò, Italy), demonstrating a mean baseline pressure of 23.2 ± 3.3 mmHg in the lymphedema cohort [15,16].

### 2.5. Statistical Analysis

Continuous variables are presented as mean ± standard deviation (SD), whereas categorical variables are expressed as frequencies and percentages [n (%)]. Normality of distribution was assessed using the Kolmogorov–Smirnov test. Differences between study groups were evaluated using Student’s *t*-test for parametric datasets and the Mann–Whitney U test for non-parametric data. Linear associations were explored using Pearson’s correlation coefficients. Differences in categorical variables were examined using the Chi-squared analysis or Fisher’s exact test, where applicable. A *p*-value < 0.05 was considered statistically significant. All statistical analyses were performed using IBM SPSS Statistics for Windows, version 29.0 (IBM Corp., Armonk, NY, USA).

## 3. Results

### 3.1. Clinical and Two-Dimensional Echocardiographic Data

Neither heart rate (70 ± 3 bpm vs. 72 ± 2 bpm, *p* = ns) nor systolic (115 ± 6 mm Hg vs. 118 ± 4 mm Hg, *p* = ns) and diastolic (78 ± 3 bpm vs. 80 ± 4 bpm, *p* = ns) blood pressure differed significantly between lymphedema patients and controls. Routine echocardiographic parameters are presented in Table 1. MAPSE did not differ significantly between patients with lymphedema and healthy controls (14.0 ± 2.8 mm vs. 12.5 ± 2.3 mm, *p* = ns). Similarly, no significant change in MAPSE was observed following MCS application (13.0 ± 4.2 mm, *p* = ns, for all). Neither patients with lymphedema nor healthy controls exhibited mitral or tricuspid regurgitation of grade ≥ 1, and no participant had evidence of valvular stenosis.

### 3.2. 3DSTE-Derived MA Dimensions and Regional LV Strains

At baseline, LV radial and circumferential strains (LV-RS and LV-CS, respectively) demonstrated a tendency toward higher values across most LV regions, with the exception of basal LV-RS. In contrast, longitudinal contraction, represented by LV longitudinal strain (LS), was reduced in the basal LV region, but tended to be increased in the midventricular and apical regions. This distinct regional contraction pattern was accompanied by reduced end-diastolic MA dimensions, whereas end-systolic mitral annular parameters remained unchanged. As a consequence, an impairment of the sphincter-like function of the MA was observed. Following MCS application, several regional LV strain values demonstrated a numerical shift toward those observed in healthy controls, with the exception of basal and apical LV-RS and midventricular and apical LV-LS. A similar directional trend was observed for end-diastolic MA dimensions. However, most of these changes did not reach statistical significance (Table 2).

### 3.3. Correlations

No correlations could be detected between MA dimensions at different phases of the cardiac cycle and basal LV strain parameters in patients with lymphedema.

### 3.4. Reproducibility of MA Parameters

Reproducibility of the MA parameters is presented in Table 3.

## 4. Discussion

Lymphedema is a chronic, progressive disorder characterized by the abnormal accumulation of protein-rich interstitial fluid within the soft tissues, predominantly affecting the extremities. Unlike primary lymphedema, which results from congenital or genetic abnormalities of the lymphatic system, secondary lymphedema is an acquired condition caused by structural damage, obstruction, or functional impairment of an otherwise anatomically normal lymphatic system. Common causes include cancer treatment, recurrent microbial infections, chronic venous insufficiency, surgical trauma, and severe obesity. Early clinical manifestations typically include persistent edema, a sensation of heaviness or tightness, localized discomfort, and restricted mobility of the affected limb. In the absence of timely treatment, chronic lymphatic stasis leads to secondary tissue remodeling, including progressive dermal thickening, subcutaneous fibrosis, and hyperkeratosis. Current standard-of-care management relies on complex decongestive therapy, in which compression-based interventions, including multilayer bandaging and specialized compression garments, play a central role in volume reduction and long-term disease control [1,2,3,4].

The mitral valve is a complex, 3D saddle-shaped structure characterized by a hyperbolic paraboloid geometry. It dynamically integrates the MA, the anterior and posterior leaflets, and the papillary muscles with their chordae tendineae. The MA is a fibrofatty ring that undergoes complex 3D deformation throughout the cardiac cycle, exhibiting both longitudinal (up-and-down) displacement and a sphincter-like narrowing and dilating pattern. This 3D motion is highly dependent on the function of the adjacent LV and, to a lesser extent, the left atrium. During systole, shortening of the basal helical LV fibers induce circumferential, sphincter-like MA contraction, which plays a pivotal role in mitral leaflet coaptation. These dynamic dimensional changes can be accurately quantified using 3DSTE. In addition, the MA undergoes systolic folding along its intercommissural axis, a phenomenon referred to as ‘saddle-deepening’, which facilitates effective leaflet coaptation while minimizing leaflet distortion and wrinkling. LV contraction also causes apical displacement of the annulus; however, this displacement is not uniform between the anterior and posterior components. Because the anterior annulus is tethered to the aortic root, it exhibits less translational movement than the posterior annulus. Consequently, the anterior aspect of the MA shifts posteriorly, reducing the distance between the anterior and posterior annular poles [17,18,19,20,21,22].

In the present study, MAPSE was used as a simple M-mode echocardiography-derived marker of longitudinal MA displacement toward the LV apex during the cardiac cycle [23,24,25]. By applying 3DSTE, we were able to assess MA dimensions throughout the cardiac cycle while simultaneously quantifying regional LV strain parameters, thereby highlighting the heterogenous nature of LV mechanics [11,12,26]. Three-dimensional echocardiography combined with STE has been extensively validated for both measurements, and normal reference values have been well-established [11,12,27,28,29,30].

The results of this study have several important implications. First, they further support the utility of 3DSTE as a comprehensive imaging modality capable of simultaneously quantifying regional LV function via strain analysis and measuring MA dimensions throughout the cardiac cycle from the same 3D echocardiographic dataset [31]. Furthermore, alongside 3D database acquisition, M-mode echocardiography allows for the concurrent measurement of longitudinal MA displacement, expressed as MAPSE, which serves as an additional robust indicator of LV function [23,24,25]. Second, findings from the routine 2D echocardiography, increased LV end-diastolic volume, reduced LV end-systolic diameter, and lower LV wall thickness were found. In lymphedema, the combination of these abnormalities is driven by chronic systemic fluid retention, leading to cardiac volume overload and compensatory eccentric remodeling. The increased volume of retained fluid enhances venous return, elevating cardiac preload; potentially promoting eccentric LV cavity dilation to accommodate the extra blood load, which directly increases the LV end-diastolic volume. In response to this stretch, the heart may recruit the Frank–Starling mechanism and trigger a compensatory hypercontractility— potentially contributing to the higher midventricular LV-RS and LV-CS observed in our cohort—which allows the myocardium to eject blood more forcefully and efficiently, ultimately leaving less residual volume at the end of contraction and thereby reducing the LV end-systolic dimensions. Finally, the lower LV wall thickness may represent a relative geometric consequence of chamber remodeling rather than true myocardial atrophy; according to Laplace’s law, as the chamber radius expands due to the volume-induced dilation without an accompanying concentric hypertrophy, the myocardial walls physically stretch and relative wall thinning [32]. Third, it was confirmed again that the presence of lymphedema is associated with alterations in regional LV contraction patterns [33]. Specifically, radial and circumferential strain values tended to be increased across most LV regions, except for basal LV radial strain. In contrast, longitudinal contraction was reduced in the basal LV region but tended to increase in the midventricular and apical segments. This characteristic contraction pattern was accompanied by decreased end-diastolic MA dimensions, preserved end-systolic parameters, and consequent impairment of MA sphincter-like functional parameters. Notably, MAPSE remained within the normal range and did not demonstrate significant abnormalities. Fourth, following MCS application, most regional LV strain parameters shifted toward values observed in healthy controls, with the exception of basal and apical LV-RS and midventricular and apical LV-LS. End-diastolic MA dimensions simultaneously demonstrated a similar trend toward values observed in healthy controls, accompanied by an improvement in MA functional parameters. In interpreting the acute, one-hour regional changes observed following compression therapy, the potential role of altered cardiac loading conditions should be considered. Although an increase in preload could theoretically modify myocardial function via the Frank–Starling mechanism, the lack of significant changes in LV end-diastolic volume in our study suggests that preload enhancement alone may not fully explain these findings. Instead, it could be hypothesized that these subtle shifts are driven by acute optimization of myocardial perfusion and a concomitant reduction in afterload. Compression therapy is known to shift peripheral venous blood toward the central circulation, potentially elevating central aortic diastolic pressure. Because myocardial perfusion occurs almost exclusively during diastole, an increase in aortic diastolic pressure could theoretically enhance coronary perfusion pressure—defined as the difference between aortic diastolic pressure and LV end-diastolic pressure. Such a hypothetical enhancement in coronary blood flow could be relevant to myocardial perfusion, mitigating subendocardial ischemia, given that the subendocardium is highly vulnerable to perfusion deficits and serves as a primary determinant of LV-LS. Consequently, a potential restoration of oxygen supply to this area might explain the numerical trends observed in myocardial strain parameters within one hour [3,4,32]. Additionally, a speculative reflex reduction in systemic vascular resistance could lower systolic wall stress and myocardial oxygen demand, potentially contributing to these acute trends. However, coronary flow, filling pressures, central hemodynamics, systemic vascular resistance, and myocardial ischemia were not directly assessed, and peripheral blood pressure remained unchanged in our cohort. Therefore these interconnected pathways remain strictly speculative hypotheses that require direct clinical validation [14,26].

The present study raises several important questions for future research. Specifically, it remains currently unknown whether these potentially favorable cardiac changes are sustained over time, whether they translate into measurable hemodynamic benefits, and whether they carry prognostic significance. Furthermore, the persistence of the observed cardiac modifications after the removal of compression stockings is to be determined, underscoring the importance of longitudinal follow-up in subsequent investigations. Consequently, further studies with extended follow-up periods are required to confirm these findings and to evaluate whether alternative imaging techniques could provide additional insights into LV and MA characterization, either by complementing or modifying the current protocol.

### Limitation Section

- The spatial and temporal resolution of 3DSTE remains inferior to that of conventional 2D echocardiography. Consequently, image quality may influence measurement accuracy and contribute to variability in derived parameters [5,6,7,8,9,10].
- Analyzing a 3D saddle-shaped annulus using 2D projections inevitably introduces geometric distortion that may result in underestimation of MA dimensions and motion parameters [17,18,19,20,21,22].
- Although 3D transesophageal echocardiography (TEE) would represent the reference imaging technique for detailed mitral valve and annular quantification and 3D reconstruction, it is semi-invasive and was not clinically justified in this cohort of asymptomatic patients with lymphedema [34]. Therefore, transthoracic 3DSTE was selected as a viable alternative due to its non-invasive nature. However, strain-based assessment of MA was not performed in this study.
- Additional myocardial strain parameters, as well as atrial and right ventricular functional indices obtainable from 3DSTE datasets, were beyond the scope of the present study and were therefore not analyzed.
- For a complete characterization of LV diastolic function, tissue Doppler echocardiography-derived E/E′ assessment should ideally have been performed, which may be considered as an important limitation.
- A key limitation of this study is the relatively small cohort size, suggesting that the study may have lacked sufficient power to detect more subtle associations and may have been susceptible to type II error. Consequently, these constraints limit the ability to draw definitive conclusions. Although the baseline characteristics remained relatively homogeneous, this manuscript should be explicitly considered as an exploratory or pilot study. Future research with larger, pre-determined sample sizes is essential to validate these preliminary findings.
- A major limitation of this study is that no correction for multiple statistical testing (such as Bonferroni or Holm–Bonferroni adjustments) was applied. Consequently, the probability of type I error may be increased, and some of the statistically significant findings might have occurred by chance. Therefore, our results should be interpreted with caution and considered exploratory and hypothesis-generating rather than confirmatory. Future studies using pre-registered, strictly controlled confirmatory protocols are needed to validate these associations.
- The study population includes patients with secondary lymphedema of diverse etiologies (chronic venous insufficiency, malignancy, cellulitis, and cancer treatment). That means that the participants did not suffer from a single underlying disease, meaning that the patient population was not entirely homogeneous. Several studies confirmed that malignant disorders and their treatments and inflammation may affect myocardial and valvular function, potentially influencing the present findings [35,36]. Moreover, chronic venous insufficiency has independent hemodynamic effects that may influence cardiac geometry, and consequently MA dynamics and should therefore be considered an important confounder [37]. These facts must be taken into account when interpreting the results.
- Moreover, sex-related differences in cardiac dimensions and functional properties may exist and should be taken into account when interpreting the findings.
- Due to logistical constraints, participant enrollment was not strictly consecutive, which may have introduced selection bias and limited external validity. However, baseline characteristics were highly comparable between the study and control groups, supporting the overall comparability of the cohorts.

## 5. Conclusions

The presented exploratory findings suggest that lymphedema may be associated with alterations in end-diastolic MA dimensions and a consequent impairment of MA function, which appear to be accompanied by specific regional LV functional abnormalities. Furthermore, one-hour MCS use was observed to exert acute, directional shifts in these imaging-derived surrogate parameters.

## Figures and Tables

**Table 1:** Two-dimensional echocardiographic data in patients with lymphedema and controls.

	Controls (n = 45)	Lymphedema Patients (n = 24)
LA diameter (mm)	35.5 ± 4.1	37.6 ± 3.9 *
LV end-diastolic diameter (mm)	46.7 ± 3.5	47.7 ± 3.9
LV end-diastolic volume (mL)	97.3 ± 20.6	108.7 ± 18.5 **
LV end-systolic diameter (mm)	31.3 ± 3.4	28.9 ± 3.3 ***
LV end-systolic volume (mL)	33.7 ± 8.4	33.4 ± 8.7
Interventricular septum (mm)	8.9 ± 1.5	8.0 ± 1.0 ***
LV posterior wall (mm)	9.2 ± 1.7	8.2 ± 1.1 ***
LV ejection fraction (%)	65.2 ± 4.1	69.8 ± 4.8 ****
E (cm/s)	77.6 ± 17.5	73.5 ± 15.3
A (cm/s)	65.6 ± 15.8	68.0 ± 15.3
E/A	1.24 ± 0.37	1.15 ± 0.38

* *p* = 0.05 vs. Controls, ** *p* = 0.03 vs. Controls, *** *p* = 0.01 vs. Controls, **** *p* = 0.0001 vs. Controls. Abbreviations: E and A = early and late diastolic transmitral flow velocities, LA = left atrial, LV = left ventricular.

**Table 2:** Comparison of three-dimensional speckle-tracking echocardiography-derived mitral annular morphological and functional parameters between patients with lymphedema and controls.

	Controls (n = 45)	Lymphedema Patients at Rest (n = 24)	Lymphedema Patients One Hour After the Use of MCS (n = 24)
**LV volumes**			
**LV-EDV (mL)**	76.8 ± 12.8	73.4 ± 16.1	79.8 ± 10.1
**LV-ESV (mL)**	34.2 ± 6.8	29.6 ± 9.2	33.3 ± 5.2
**LV-EF (%)**	56.3 ± 7.2	60.5 ± 6.1 *	58.5 ± 4.7
**LV strains**			
**basal LV-RS (%)**	33.1 ± 16.2	29.0 ± 11.2	28.4 ± 18.0
**mid LV-RS (%)**	27.9 ± 10.3	34.3 ± 8.6 **	30.3 ± 12.1
**apical LV-RS (%)**	17.5 ± 8.0	21.5 ± 12.5	23.1 ± 13.9
**basal LV-CS (%)**	−24.6 ± 6.1	−26.5 ± 6.9	−24.7 ± 6.1
**mid LV-CS (%)**	−26.2 ± 5.6	−31.9 ± 5.6 ***	−28.9 ± 4.4
**apical LV-CS (%)**	−32.5 ± 10.3	−37.0 ± 9.1	−34.2 ± 7.9
**basal LV-LS (%)**	−22.4 ± 5.2	−18.3 ± 4.7 ****	−20.2 ± 4.1
**mid LV-LS (%)**	−14.6 ± 3.3	−16.2 ± 4.5	−16.1 ± 2.9 **
**apical LV-LS (%)**	−16.9 ± 5.9	−18.3 ± 5.3	−18.8 ± 3.1
**MA morphological parameters**			
**MAD-D (cm)**	2.43 ± 0.39	2.20 ± 0.36 †	2.28 ± 0.41
**MAA-D (cm^2^)**	7.41 ± 2.18	6.04 ± 1.53 ††	6.88 ± 2.03
**MAP-D (cm)**	10.25 ± 1.52	9.33 ± 1.17 †††	10.00 ± 1.53
**MAD-S (cm)**	1.59 ± 0.40	1.59 ± 0.41	1.55 ± 0.32
**MAA-S (cm^2^)**	3.35 ± 1.20	3.73 ± 1.30	3.85 ± 1.12
**MAP-S (cm)**	6.90 ± 1.10	7.41 ± 1.21	7.44 ± 1.05
**MA functional parameters**			
**MAFAC (%)**	53.19 ± 15.74	38.33 ± 14.65 ††††	41.22 ± 17.49 †††††
**MAFS (%)**	33.90 ± 14.95	27.81 ± 12.33	31.11 ± 12.21

* *p* = 0.05 vs. Controls, ** *p* = 0.03 vs. Controls, *** *p* = 0.01 vs. Controls, **** *p =* 0.02 vs. Controls, † *p* = 0.02 vs. Controls, †† *p* = 0.01 vs. Controls, ††† *p* = 0.02 vs. Controls, †††† *p* = 0.0004 vs. Controls, ††††† *p* = 0.01 vs. Controls. Abbreviations: LV = left ventricular, EDV = end-diastolic volume, ESV = end-systolic volume, EF = ejection fraction, RS = radial strain, CS = circumferential strain, LS = longitudinal strain, mid = midventricular, MA = mitral annulus, MAD = mitral annular diameter, MAA = mitral annular area, MAP = mitral annular perimeter, D = end-diastolic, S = end-systolic, MAFAC = mitral annular fractional area change, MAFS = mitral annular fractional shortening, MCS = medical compression stocking.

**Table 3:** Intra- and interobserver variability for three-dimensional speckle-track echocardiography-derived mitral annular dimensions.

	Intraobserver Agreement		Interobserver Agreement	
	Mean ± 2SD Difference in Values Obtained by 2 Measurements of the Same Observer	ICC Between Measurements of the Same Observer	Mean ± 2SD Difference in Values Obtained by 2 Observers	ICC Between Independent Measurements of 2 Observers
End-diastolic MAD	0.03 ± 0.09 cm	0.96 (*p* < 0.0001)	0.03 ± 0.17 cm	0.95 (*p* < 0.0001)
End-diastolic MAA	−0.04 ± 0.51 cm^2^	0.96 (*p* < 0.0001)	0.04 ± 0.34 cm^2^	0.94 (*p* < 0.0001)
End-diastolic MAP	−0.04 ± 0.64 cm	0.96 (*p* < 0.0001)	−0.05 ± 0.53 cm	0.94 (*p* < 0.0001)
End-systolic MAD	−0.03 ± 0.06 cm	0.95 (*p* < 0.0001)	0.03 ± 0.12 cm	0.96 (*p* < 0.0001)
End-systolic MAA	−0.04 ± 0.11 cm^2^	0.98 (*p* < 0.0001)	−0.04 ± 0.78 cm^2^	0.97 (*p* < 0.0001)
End-systolic MAP	0.06 ± 0.55 cm	0.93 (*p* < 0.0001)	0.05 ± 0.55 cm	0.94 (*p* < 0.0001)

Abbreviations: ICC = interclass correlation coefficient, MAD = mitral annular diameter, MAA = mitral annular area, MAP = mitral annular perimeter, SD = standard deviation.

## Data Availability

The data presented in this study are available on request from the corresponding author due to local restrictions.
